# Hydroxyurea-Increased Fetal Hemoglobin Is Associated with Less Organ Damage and Longer Survival in Adults with Sickle Cell Anemia

**DOI:** 10.1371/journal.pone.0141706

**Published:** 2015-11-17

**Authors:** Courtney D. Fitzhugh, Matthew M. Hsieh, Darlene Allen, Wynona A. Coles, Cassie Seamon, Michael Ring, Xiongce Zhao, Caterina P. Minniti, Griffin P. Rodgers, Alan N. Schechter, John F. Tisdale, James G. Taylor

**Affiliations:** 1 Molecular and Clinical Hematology Branch, NHLBI/NIDDK, NIH, Bethesda, Maryland, United States of America; 2 Hematology Branch, NHLBI, NIH, Bethesda, Maryland, United States of America; 3 Office of the Clinical Director, NIDDK, NIH, Bethesda, Maryland, United States of America; 4 Intramural Research, NIDDK, NIH, Bethesda, Maryland, United States of America; 5 Molecular Medicine Branch, NIDDK, NIH, Bethesda, Maryland, United States of America; Sickle Cell Unit, JAMAICA

## Abstract

**Background:**

Adults with sickle cell anemia (HbSS) are inconsistently treated with hydroxyurea.

**Objectives:**

We retrospectively evaluated the effects of elevating fetal hemoglobin with hydroxyurea on organ damage and survival in patients enrolled in our screening study between 2001 and 2010.

**Methods:**

An electronic medical record facilitated development of a database for comparison of study parameters based on hydroxyurea exposure and dose. This study is registered with ClinicalTrials.gov, number NCT00011648.

**Results:**

Three hundred eighty-three adults with homozygous sickle cell disease were analyzed with 59 deaths during study follow-up. Cox regression analysis revealed deceased subjects had more hepatic dysfunction (elevated alkaline phosphatase, Hazard Ratio = 1.005, 95% CI 1.003–1.006, p<0.0.0001), kidney dysfunction (elevated creatinine, Hazard Ratio = 1.13, 95% CI 1.00–1.27, p = 0.043), and cardiopulmonary dysfunction (elevated tricuspid jet velocity on echocardiogram, Hazard Ratio = 2.22, 1.23–4.02, p = 0.0082). Sixty-six percent of subjects were treated with hydroxyurea, although only 66% of those received a dose within the recommended therapeutic range. Hydroxyurea use was associated with improved survival (Hazard Ratio = 0.58, 95% CI 0.34–0.97, p = 0.040). This effect was most pronounced in those taking the recommended dose of 15–35 mg/kg/day (Hazard Ratio 0.36, 95% CI 0.17–0.73, p = 0.0050). Hydroxyurea use was not associated with changes in organ function over time. Further, subjects with higher fetal hemoglobin responses to hydroxyurea were more likely to survive (p = 0.0004). While alkaline phosphatase was lowest in patients with the best fetal hemoglobin response (95.4 versus 123.6, p = 0.0065 and 96.1 versus 113.6U/L, p = 0.041 at first and last visits, respectively), other markers of organ damage were not consistently improved over time in patients with the highest fetal hemoglobin levels.

**Conclusions:**

Our data suggest that adults should be treated with the maximum tolerated hydroxyurea dose, ideally before organ damage occurs. Prospective studies are indicated to validate these findings.

## Introduction

Homozygous sickle cell disease (HbSS) leads to polymerization of deoxygenated sickle hemoglobin within rigid red blood cells (RBC) which then occlude microvasculature, resulting in acute complications, chronic organ damage, and premature death [1]. Many deaths today occur in young adults. A 1994 adult mortality analysis showed 64% of deaths occurred in patients without end-organ damage, 18% with organ dysfunction, and 22% during a painful crisis [2]. This contrasts a four decade observational study, where most deaths were associated with irreversible end organ damage [3]. Thus, organ dysfunction is an important risk factor for death, even though it is unclear if detectable organ damage is infrequent or common [4–9].

In the Multicenter Study of Hydroxyurea (MSH), hydroxyurea decreased acute complications and transfusions [10]. The MSH did not specifically report fetal hemoglobin (HbF) responses, although other reports have shown that hydroxyurea largely exerts its effect by increasing HbF and inhibiting RBC sickling [11,12]. While Powars and colleagues did not find a linear trend between HbF levels and morbidity, the only markers of organ damage that they evaluated were stroke and avascular necrosis, but not liver, kidney, heart, or pulmonary dysfunction [13]. Long-term hydroxyurea studies suggest improved survival [7,8,14]. Conversely, studies from the post-hydroxyurea approval era report patients continue to die by the fifth decade [4–6,9,15,16]. Health database studies have shown no change in mortality since hydroxyurea approval [9,17], and recent studies have shown no association between current hydroxyurea use and mortality [16,18,19]. However, the effects of dose and HbF response were not analyzed in these studies, and only 7–42% of patients were prescribed hydroxyurea at the time of death [4–6,15,16]. We hypothesized that these conflicting findings are attributable to dose-dependent effects which have not been previously analyzed. Thus, we performed a single institution, retrospective analysis to determine the effect of hydroxyurea dose on HbF response, organ damage, and survival.

## Methods

### Patient Population

Subjects enrolled in the screening study between 2001 and 2010 were included; data were collected through 2012. Entry criteria (18 years of age or older and a diagnosis of sickle cell disease) and recruitment are described elsewhere [19,20]. Clinical, laboratory, and echocardiographic evaluations were performed during steady state at enrollment and follow-up visits every two years. The study was approved by the Institutional Review Board of the National Heart, Lung, and Blood Institute and all subjects gave written informed consent.

### Cause of Death Assignment

Mortality data were updated every two years via patient contact or inquiries to the Social Security Death Index. All death certificates were requested. A consent waiver was issued by the Office of Human Subjects Protection, National Institutes of Health to obtain medical records surrounding the time of death and the three months preceding death from hospitals where subjects died. Cause of death was assigned by two investigators independently (CF and MH).

### Clinical, Laboratory, and Echocardiographic Data

Sickle cell disease (SCD) phenotypes were assigned based upon DNA sequencing and/or high performance liquid chromatography [21]. Age at death was confirmed by death certificate, and age of living patients was assigned as the age at last follow-up. Outpatient studies were compared between the time of enrollment (first visit) and at most recent follow-up (last visit). Data obtained from outside hospitals near the time of death were also included. Mean corpuscular volume (MCV) was evaluated in all patients because it increases in response to hydroxyurea. For each subject, maximum HbF and MCV were defined as the highest observed values, and mean maximum HbF and MCV were defined as the mean calculation of these values. In some analyses, the patients were divided into quartiles based on mean maximum HbF concentration (HbF ≤6.5% defined the lowest quartile and HbF >19.9% defined the highest quartile).

### Hydroxyurea Exposure and Dose Determination

While hydroxyurea treatment was recommended by NIH investigators for appropriate indications at enrollment, treatment and dosing decisions were made at the discretion of each subject’s primary provider. The NIH Biomedical Translational Research Information System (BTRIS) was used to electronically screen notes for the word “hydroxyurea” and synonyms. Computer-assisted review facilitated the extraction of doses from over 20,000 records. Hydroxyurea exposure was positive if the subject had documentation of ever receiving hydroxyurea and maximum hydroxyurea dose was recorded. Duration of hydroxyurea treatment was not able to be determined using these records, as most subjects did not receive routine care at NIH. When laboratory data suggested treatment without electronic documentation, charts were manually reviewed to determine exposure status and dose. The recommended hydroxyurea dose is between 15 and 35 mg/kg/day [22].

### Statistical Analysis

Descriptive statistics compared groups based on survival, hydroxyurea exposure, and maximum HbF response. Comparisons were performed using t-tests, Wilcoxon rank-sum tests, and Chi-square tests, where appropriate. Means and standard deviations are reported, if not otherwise specified. Cox proportional hazard regression using age as the time-scale was used to relate survival and hydroxyurea exposure with laboratory values and other potential co-variates. All analyses were performed using SAS (version 9.1.3; SAS Institute, Cary, NC) and JMP (version 8.0; SAS Institute, Cary, NC). The significance level was set at p<0.05. All statistical testing was two-sided and no multiplicity was corrected.

## Results

### Characteristics of Subjects

The 383 subjects had a median enrollment age of 31.0 (range 18–74) years with equal gender proportions (184 subjects, 48% male), and sixty-six percent (n = 253) had ever been treated with hydroxyurea. Median follow-up was 2.6 (0.1–11.7) years. Fifty-nine subjects (15%) were deceased with a median age at death of 46.0 (18–74) years; 46.0 years for men and 44.5 years for women.

### Causes of Death

Among the entire cohort, 59 deaths occurred at 20 outside hospitals. Medical records for 16 deceased patients were received from 7 hospitals. Death certificates were obtained for 44 (75%), and autopsy reports were available for 19 (32%). The most common identifiable causes of death were pulmonary (N = 11, Table 1). Other common known causes of death were sickle cell crisis (N = 7), infection (N = 6), and cardiac (N = 5). Cause of death was unknown for nine and was listed as SCD on the death certificates in another eight.

**Table 1:** Causes of Death in Patients with Homozygous Sickle Cell Disease.

Cause of Death	Subgroup N	Total N (%)
Pulmonary		11 (19%)
Pulmonary Hypertension/Cor Pulmonale^a^	7	
Pulmonary Embolism^a^	2	
Acute Chest Syndrome	2	
Sickle Cell Disease Not Otherwise Specified		8 (15%)
Sickle Cell Crisis		7 (12%)
Infection		6 (10%)
Sepsis	3	
Pneumonia	2	
Endocarditis	1	
Cardiac		5 (8%)
Congestive Heart Failure^a^	3	
Hypertensive Cardiovascular Disease	1	
Atherosclerotic Cardiovascular Disease	1	
Narcotic Toxicity^a^		4 (7%)
Gastrointestinal		4 (7%)
Bleeding	2	
Liver Failure	1	
Acute Pancreatitis	1	
Cerebrovascular		3 (5%)
Intracranial Hemorrhage	2	
Stroke^a^	1	
Other^b^		6 (10%)
Unknown		9 (15%)

^a^More than one cause of death was assigned to 4 patients

^b^Other: multi-organ failure (1); trauma (1); subglottic stenosis (1); acute myelogenous leukemia (1); hemolytic transfusion reaction (1); cocaine toxicity (1)

### Deceased HbSS Subjects have More Laboratory Evidence of Organ Injury

First we compared characteristics at enrollment where deceased subjects (n = 59) were significantly older at enrollment (41.8 versus 32.5 years, p = 0.0067), had lower maximum HbF (p = 0.0044), were less likely to have taken hydroxyurea (56% versus 68%, p = 0.040, hazard ratio 0.58, 95% confidence interval 0.34, 0.97), and had a lower proportion prescribed hydroxyurea within the recommended dosage range (29% versus 46%, p = 0.0039, Table 2). There was no significant difference in MCV between living and deceased patients. Mean duration between data collection at enrollment and time of death was 997.0 +/- 935.0 days for the deceased subjects. Univariate comparison of initial evaluation and most recent follow-up showed deceased patients had significantly worse white blood cell count, hepatic (albumin, alkaline phosphatase, and direct bilirubin), renal (creatinine, uric acid), and cardiopulmonary (tricuspid regurgitant velocity, TRV), parameters (p<0.05). There were no differences in hemoglobin or lactate dehydrogenase (LDH) between living and deceased subjects at enrollment. At the last visit, while hemoglobin concentrations remained similar, deceased subjects had significantly lower HbF (6.1 versus 10.8%, p<0.0001) and higher LDH (530.6 versus 378.1U/L, p = 0.0159).

**Table 2:** Characteristics Regarding Hydroxyurea in Living and Deceased Subjects with HbSS.

Variable	Alive (N = 324)	Deceased (N = 59)	P-value
Age at Enrollment (years)	32.5 ± 11.3	41.8 ± 12.1	0.0067^a^
Follow-up Time (years)	7.1 ± 3.3	3.7 ± 2.7	<0.0001^b^
Gender [N (%)]			0.25^a^
Male	169 (52%)	30 (51%)	
Female	155 (48%)	29 (49%)	
Hydroxyurea Exposure [N (%)]			0.040^a^
Yes	220 (68%)	33 (56%)	
No	104 (32%)	26 (44%)	
Hydroxyurea Dosage Class [N (%)]			-
None	104 (32%)	26 (44%)	-
<15 mg/kg/d	39 (12%)	6 (10%)	0.53
15–35 mg/kg/d	149 (46%)	17 (29%)	0.0039
>35 mg/kg/d^c^	22 (7%)	3 (5%)	0.97
Yes but Hydroxyurea Dose Unknown	10 (3%)	7 (12%)	0.12^a^
Mean Maximum HbF (%)^d^	14.3 ± 9·5	11.3 ± 11·4	0.0044^a^
Mean Maximum MCV (fL)^d^	102.7 ± 15·2	104.2 ± 16.5	0.57^a^

^a^Cox regression with age as the time scale

^b^t-test

^c^Hydroxyurea doses >35 mg/kg/d are not FDA-approved for the general management of patients with sickle cell anemia and were usually used in patients in preparation for hematopoietic stem cell transplantation.

^d^Mean maximum HbF and MCV are defined as the mean calculation of the highest values observed for each subject.

Multivariable Cox regressions using age as the time-scale were performed to further identify baseline factors that are associated with survival time. This confirmed an independent association between organ dysfunction and survival (Table 3). Higher alkaline phosphatase (p<0.0001) as well as white blood cells (WBC, p = 0.048) creatinine (p = 0.043), and TRV (p = 0.0082) at enrollment were independently associated with death. Subjects who administered a dose between 15 and 35 mg/kg/day were more likely to be alive than subjects who never took hydroxyurea (p = 0.0050, hazard ratio 0.36 with 95% confidence interval (0.17, 0.73)). Regression models and parameter estimates are given in Table 3.

**Table 3:** Cox Regression Analysis of Variables Associated with Survival for Subjects with HbSS.

Variable^a^	Hazard Ratio, 95% Confidence Interval	P-value
**Enrollment Visit**		
Alkaline Phosphatase	1.005 (1.003, 1.006)	<0.0001
White Blood Cell Count	1.08 (1.00, 1.17)	0.048
Creatinine	1.13 (1.00–1.27)	0.043
Tricuspid Regurgitant Velocity	2.22 (1.23–4.02)	0.0082
Hydroxyurea Dose <15 mg/kg/day	0.73 (0.26–2.05)	0.55
Hydroxyurea Dose 15–35 mg/kg/day	0.36 (0.17–0.73)	0.0050
Hydroxyurea Dose >35 mg/kg/day	0.72 (0.20–2.55)	0.61
Hydroxyurea Dose Unknown	2.41 (0.96–6.09)	0.063

^a^Input variables: age, hydroxyurea exposure, hydroxyurea dose, dose group, maximum fetal hemoglobin, hemoglobin, white blood cell count, alkaline phosphatase, total bilirubin, albumin, creatinine, ejection fraction, and tricuspid regurgitant velocity. The input variables were selected based on univariate analysis results if they were associated either with mortality or hydroxyurea use. The final model is shown in the table and is obtained through backward stepwise model selection and includes variables associated with hydroxyurea use if they were significant at the 0.10 level. The hazard ratio units represent increase per one unit change of the factor. Hydroxyurea dose groups are compared to no hydroxyurea.

### Hydroxyurea Dose Is Associated with Survival

Because hydroxyurea was associated with living status (Table 2), and because organ damage was associated with early mortality, we sought to examine the effect of its dosing on survival and on markers of organ damage. Among 66% of HbSS subjects taking hydroxyurea (N = 253), only 166 (66%) were on the recommended dose (Table 4). Importantly, another 45 (18%) were on a dose below 15 mg/kg/day. The mean hydroxyurea dose was 21.5 mg/kg/day. The average maximum HbF was greater (17.3 versus 7.2, p<0.0001) and maximum MCV higher (109.4 versus 90.2, p<0.0001) in subjects who took hydroxyurea.

**Table 4:** Characteristics at Enrollment and Survival Status Based On Hydroxyurea Status and Fetal Hemoglobin Quartiles in Patients with HbSS.

Variable	No Hydroxyurea (N = 130)	Any Hydroxyurea (N = 253)	HbF Lower 25% (N = 99)	HbF Upper 25% (N = 96)
Age (Years)^a^	35.6±12.9	33.1±11.3	34.8 ± 13.4	35.4 ± 11.36
Follow-up Time (Years)^a^	6.3 ± 3.7	6.8 ± 3.3	5.3 ± 3.3	7.4 ± 3.3**
Gender [N (%)]^b^				
Male	72 (55%)	126 (50%)	53 (54%)	47 (49%)
Female	58 (45%)	127 (50%)	46 (46%)	49 (51%)
Survival Status [N (%)]				
Alive	104 (80%)	220 (87%)	70 (71%)	82 (85%)*
Deceased	26 (20%)	33 (13%)	29 (29%)	14 (15%)
Hydroxyurea Exposure [N (%)]^b^				
Yes	0 (0%)	253 (100%)^N/A^	33 (33%)	93 (97%)**
No	130 (100%)	0 (0%)	66 (67%)	3 (3%)
Mean Maximum Hydroxyurea Dose (mg/kg/d)^d^	0	21.5 ± 11.9^N/A^	6.0 ± 10.3	23.0 ± 11.2**
Hydroxyurea Dosage Class [N (%)]^b^				
None	130 (100%)	0 (0%)^N/A^	66 (67%)	3 (3%)**
<15 mg/kg/d	0 (0%)	45 (18%)	11 (11%)	10 (10%)
15–35 mg/kg/d	0 (0%)	166 (66%)	18 (18%)	72 (75%)
>35 mg/kg/d^e^	0 (0%)	25 (10%)	2 (2%)	8 (8%)
Yes but Hydroxyurea Dose Unknown	0 (0%)	17 (7%)	2 (2%)	3 (3%)
Mean Maximum HbF (%)^d^ ^f^	7.2 ± 5.4	17.3 ± 9.9^##^	3.1 ± 1.7	27.7 ± 6.6^N/A^
Mean Maximum MCV (fL)^a^ ^f^	90.2 ± 9.2	109.4 ± 13.8^##^	90.7 ± 9.8	118.2 ± 12.3**
Mean HbF (%)^a^	6.7 ± 5.2	9.2 ± 6.7^#^	2.8 ± 1.9	12.7 ± 7.8^N/A^
Mean MCV (fL)^a^	88.2 ± 9.1	96.4 ± 10.8^##^	87.5 ± 8.7	97.6 ± 11.3**

^a^t-test

^b^Chi-square test

^c^Cox regression with age as the time-scale

^d^Wilcoxon rank-sum test

^N/A^Not applicable

^e^Hydroxyurea doses >35 mg/kg/d are not FDA-approved for the general management of patients with sickle cell anemia and were usually used in patients in preparation for hematopoietic stem cell transplantation.

^f^Mean maximum HbF and MCV are defined as the mean calculation of the highest values observed for each subject.

*p<0.001 when comparing HbF Lower 25% to HbF Upper 25% groups

**p<0.0001 when comparing HbF Lower 25% to HbF Upper 25% groups

^#^p<0.001 when comparing No Hydroxyurea to Any Hydroxyurea groups

^##^p<0.0001 when comparing No Hydroxyurea to Any Hydroxyurea groups

Subjects receiving hydroxyurea had significantly higher HbF (9.2 versus 6.7%, p = 0.0003 and 12.1 versus 6.5%, p<0.0001) and MCV (96.4 versus 88.2fL, p<0.0001, and 99.3 versus 87.4fL, p<0.0001, Table 5) at their initial visit and most recent visit, respectively, as compared to those not taking hydroxyurea, suggesting prolonged treatment. However, hemoglobin was not different between groups at either visit (88 versus 90g/L at initial visit, p = 0.36 and 89 versus 88g/L at most recent follow-up, p = 0.62 in hydroxyurea versus no hydroxyurea groups). Further, while absolute neutrophil count (ANC) was lower at the last visit with hydroxyurea (5.6 versus 6.1, p = 0.075 at first visit and 4.9 versus 6.3, p<0.0001 at last visit in subjects taking versus not taking hydroxyurea), the value was considerably above the ANC of 2,000u/L which defines the maximum tolerated dose [10].

**Table 5:** Comparison of Hematologic and Organ Function Parameters at Enrollment and Last visits in Patients with HbSS Based on Hydroxyurea Status and Fetal Hemoglobin Quartile.

	Enrollment Visit				Last Visit			
Variable	No HU (N = 130)	Any HU (N = 253)	Low HbF (N = 99)	High HbF (N = 96)	No HU (N = 130)	Any HU (N = 253)	Low HbF (N = 99)	High HbF (N = 96)
**Hematologic**								
White Blood Cell Count (K/uL)	11.4	10.4^#^	12.2	9.9***	11.4	8.8^###^	11.5	7.7***
ANC (K/uL)	6.1	5.6	6.6	5.2*	6.3	4.9^###^	6.4	4.3***
Hemoglobin (g/L)	90	88	87	90	88	89	86	91*
Hematocrit (%)	26.4	25.6	25.6	25.9	25.9	25.4	25.6	26.1
HbF (%)	6.7	9.2^##^	2.8	12.7***	6.5	12.1^###^	2.9	17.8***
MCV (fL)	88.2	96.4^###^	87.7	97.6***	87.4	99.3^###^	87.9	103.9***
Platelet Count (K/uL)	410.8	400.9	431.2	364.0*	379.7	353.2	400.6	319.2**
Reticulocyte (%)	9.5	10.4	10.1	9.3	9.7	9.3	10.5	8.1*
ARC (K/uL)	259.0	267.2	265.8	236.9	259.4	223.0^#^	268.1	193.6**
**Hepatic**								
Albumin (g/L)	41	40	40	41	39	39	39	39
Alkaline Phosphatase (U/L)^a^ ^,^ ^b^	112.0	112.2	123.6	95.4*	110.3	109.3	113.6	96.1*
ALT (U//L)^a^ ^,^ ^b^	30.8	29.7	33.4	27.0*	34.8	34.3	41.4	32.3
AST (U/L)^a^ ^,^ ^b^	45.5	45.2	50.5	42.5*	47.2	45.3	54.6	41.5*
Total Bilirubin (mg/dL)^a^ ^,^ ^b^	2.8	3.3^#^	3.5	2.9	2.7	2.7	3.2	2.2**
Direct Bilirubin (mg/dL)^a^ ^,^ ^b^	0.5	0.6	0.7	0.5	0.5	0.7	0.6	0.5
**Renal**								
Creatinine (mg/dL)^a^ ^,^ ^b^	1.2	0.8^#^	1.3	0.8*	1.4	1.1	1.3	1.0
Phosphorus (mg/dL)^a^	4.2	4.1	4.3	4.1	3.9	3.8	4.0	3.8
Uric Acid (mg/dL)^a^	6.3	6.3	6.6	6.3	6.2	5.9	6.7	5.8*
**Cardiopulmonary**								
Ejection Fraction^c^ (%)	58.4	58.5	57.5	58.9	59.5	59.6	58.4	59.6
TRV^c^ (m/s)	2.5	2.6^#^	2.6	2.6	2.6	2.7	2.7	2.7
NT-ProBNP^d^ (pg/mL)^b^	91.9	116.6	114.9	123.9	109.0	173.6	138.5	236.9
**Other**								
Lactate Dehydrogenase (U/L) ^a^ ^,^ ^b^	376.9	389.6	399.8	382.5	385.7	408.4	409.3	426.4

Abbreviations: HU, hydroxyurea; Low HbF, maximum fetal hemoglobin within the lowest quartile; High HbF, maximum fetal hemoglobin within the highest quartile; ANC, absolute neutrophil count; MCV, mean corpuscular volume; ARC, absolute reticulocyte count; ALT, alanine aminotransferase; AST, aspartate aminotransferase; TRV, tricuspid regurgitant velocity; NT-ProBNP, brain natriuretic peptide

^a^Conversion factor from U/L to μkat/L is 0.017; conversion factor from mg/dL to μmoL/L is 17.1

^b^Wilcoxon rank-sum test, other p-values are from t-tests if not otherwise specified

^c^Ejection fraction and TRV were reported in 345 subjects at first visit and 261 subjects at last visit

^d^NT-ProBNP levels are only reported in patients with a creatinine <1.0mg/dL, (N = 65 No HU, 165 any HU, 21 low HbF, 12 high HbF)

^#^p<0.05 when comparing No Hydroxyurea to Any Hydroxyurea groups

^##^p<0.001 when comparing No Hydroxyurea to Any Hydroxyurea groups

^###^p<0.0001 when comparing No Hydroxyurea to Any Hydroxyurea groups

*p<0.05 when comparing HbF Lower 25% to HbF Upper 25% groups

**p<0.001 when comparing HbF Lower 25% to HbF Upper 25% groups

***p<0.0001 when comparing HbF Lower 25% to HbF Upper 25% groups

### No Difference in Organ Function over Time Based on Hydroxyurea Exposure or Hydroxyurea Dose

To assess whether hydroxyurea exposure was associated with decreased organ damage, we next compared laboratory studies at enrollment to most recent follow-up between patients who had versus had never taken hydroxyurea. At the first visit, total bilirubin was greater (3.3 versus 2.8mg/dL, p = 0.018), creatinine lower (0.8 versus 1.2mg/dL, p = 0.0015), and TRV higher (2.6 versus 2.5m/s, p = 0.025) in patients taking hydroxyurea (Table 5). Conversely, at the last visit, there were no significant differences in markers of organ function in patients taking versus not taking hydroxyurea. Therefore, there was no apparent difference in organ dysfunction over time based solely on whether patients had received hydroxyurea. Subjects were then divided into groups based on whether their dose was below (0–15 mg/kg/day) versus at or above the recommended dose (15–35 mg/kg/day). At the first visit, creatinine was lower in patients who took the recommended dose (0.7 versus 1.2mg/dL, p = 0.002). However, there were no significant differences in markers of organ function at the last visit. Therefore, hydroxyurea dose per se did not lead to differences in organ function over time.

### Higher HbF Quartiles Are Associated with Improved Survival, Higher Hydroxyurea Dosing, and Less Organ Injury over Time

To evaluate whether increased hydroxyurea-induced HbF is necessary to prevent organ damage and improve survival, subjects were divided into quartiles based on maximum HbF (Table 4). While age was similar, subjects with all of the higher HbF quartiles were more likely to be alive. While 71% of patients in the lowest HbF quartile were living, 90% in the second quartile, 91% in the third quartile, and 86% in the highest quartile were living (p = 0.0004). This corresponded to a hazard ratio for the second quartile compared to the lowest quartile of 0.330, 95% CI (0.156, 0.698), p = 0.0037, hazard ratio for the third quartile compared to the lowest quartile of 0.191, 95% CI (0.077, 0.473), p = 0·0003, and hazard ratio for the highest quartile compared to the lowest quartile of 0.303, 95% CI (0.151, 0.610), p = 0·0008).

Almost all subjects within the highest HbF group were assigned to the hydroxyurea group (97%) as compared to 33% in the lowest group (p<0.0001, Table 4). Further, 75% of the highest HbF group were treated with recommended doses compared to only 18% in the lowest group (p<0.0001). In contrast, 67% of patients in the low HbF group were not taking hydroxyurea. Median hydroxyurea dosages were 23.0 in the highest quartile, 15.9 in the third quartile, 12.3 in the second quartile, and 5.9 mg/kg/day in the lowest quartile, (p<0.0001), with a mean maximum HbF of 27.7, 15.7, 9.0, and 3.1% (p<0·0001) and a mean HbF of 12.7, 10.9, 7.1, and 2.8% (p<0.0001) at the time of enrollment, respectively.

Mean HbF at the last visit was 17.8% in the highest HbF quartile, which showed an absolute increase of 5.1% as compared to the initial visit (Table 5). At first and last visit, alkaline phosphatase was significantly lower (95.4 versus 123.6, p = 0.0065 and 96.1 versus 113.6, p = 0.041, respectively) in patients with the highest HbF. While ALT was also significantly lower at the first visit (27.0 versus 33.4U/L, p = 0·0086), there was no significant difference at the last visit (32.3 versus 41.4U/L, p = 0.076). Again while creatinine was also significantly lower at the first visit (0.8 versus 1.3mg/dL, p = 0.0108), there was no significant difference at the most recent visit (1.0 versus 1.3mg/dL, p = 0.080) in the highest HbF group. Interestingly, there were no differences in ejection fraction, TRV, or brain natriuretic peptide (NT-proBNP) based on HbF quartile. Therefore, we did not find a clear association between optimized HbF response and decreased organ damage in this retrospective analysis.

## Discussion

Median age at death for SCD has not changed since 1994, with a median age at death of 46 years in our cohort [2]. In our modern cohort, where cause of death was determined for 85% of subjects, the most prevalent attribution was pulmonary disease, which led us to hypothesize that organ dysfunction might predict mortality. We have identified parallel determinants of mortality in this cohort, although neither is associated with specific causes of death. First, laboratory studies indicating a more severe phenotype, including lower HbF and higher WBC and LDH values, were significantly different prior to death compared to those still living [2,8]. Hemoglobin was not different between groups, suggesting that these parameters might reflect chronic tissue damage and not increased hemolysis [23]. More importantly, we document hepatic injury at an initial visit among subjects who later died (Table 3), in contrast to studies where organ damage was not routinely identified before death [2,5]. Deceased patients also had a significantly higher TRV, which was not surprising since 132 HbSS patients included in the study were reported in the original study reporting high TRV as a mortality risk factor [20]. While organ injury did not correlate with causes of death, this observational cohort study limits definitive evaluation of these relationships. Overall, laboratory screening may help to identify candidates for treatment intensification.

This study also demonstrates a 66% treatment rate, although a surprising 18% were treated below the recommended dose range. This is an improvement over prior reports where less than 50% of clinicians and only 16% of hematologists prescribed hydroxyurea [24,25]. Further, while a follow-up study reported a significant increase in HbF from baseline to 2 years after hydroxyurea treatment [26], the pivotal study which led to hydroxyurea’s FDA-approval did not report HbF levels. As a result, there has been a shift in the field away from investigation of HbF induction. This lack of definitive mechanisms by which hydroxyurea exerts its benefit likely also contributes to its limited use in practice.

Because hydroxyurea could delay end organ damage, we also analyzed its effect upon organ damage and mortality by both treatment and hydroxyurea dose. Our study shows an association between the recommended hydroxyurea dose and survival. To our knowledge, this is the first study to demonstrate that doses below the recommended starting dose may not confer a survival benefit (Table 3). Long term studies have shown a similar relationship between lower mortality and hydroxyurea titration to a maximally tolerated dose [7,8,14]. Contemporary studies reporting early deaths in the post-hydroxyurea approval era were limited by a small proportion administered hydroxyurea [4–6,9,15,16]. Recent studies reported no survival difference based on hydroxyurea use [16,18]; however, dosing was not mentioned. Similar to these studies, we report a young median age at death. Though the majority had administered hydroxyurea, mean dose was only 21.5 mg/kg/day. The insufficiently suppressed ANC and a lack of hemoglobin rise suggest that hydroxyurea dosing could be increased to a maximally tolerated dose as is frequently recommended by pediatric hematologists in the United States [27]. Alternatively, patients in all three upper quartiles of HbF were more likely to be alive. Therefore, our data suggest that even moderate increases, and not necessarily maximum HbF induction, may improve survival in patients with HbSS. It is also interesting to note that while patients started on optimal hydroxyurea doses presumably had more severe disease, survival was actually lower in this patient group.

We next wanted to assess whether optimized hydroxyurea dosing may improve organ function. Our data do not support improved organ function based solely on hydroxyurea presence. A randomized study of fixed dose hydroxyurea (20 mg/kg/day versus placebo) in infants with HbSS showed improvement in markers of splenic function, suggesting that even modest HbF increases can be beneficial [28]. This fixed dose is comparable to our mean hydroxyurea dose. However, there was no significant improvement in splenic function by radionuclide scan or in glomerular filtration rate in the hydroxyurea treated infants. Conversely, two pediatric studies where patients were treated with maximally tolerated doses of hydroxyurea reported splenic regeneration [29,30]; one confirming that 25% HbF was more likely to be associated with some splenic regeneration as compared to 14% HbF [30]. An additional study reported that in children on hydroxyurea, those with sickle retinopathy had significantly lower HbF levels as compared to those without retinopathy (HbF 9 versus 16%, p = 0.005, respectively) [31]. These data suggest that patients might also benefit from further dose escalation to maximize preservation of organ function.

To further assess whether hydroxyurea-induced HbF reduced organ injury, we compared laboratory values from the highest and lowest HbF quartiles. While we found a significantly lower creatinine and ALT at the first visit, the only marker of organ damage that was consistently lower during both visits was alkaline phosphatase. Hydroxyurea decreases deaths associated with liver dysfunction [8]. While the maximum HbF was 27.7% in the high group, values measured strictly at enrollment and last follow-up ranged from 12.7 to 17.8%, which may not have been sufficient to prevent organ damage or reverse organ dysfunction. It is possible that HbF approaching 30% may be crucial to prevent organ injury and vascular damage [2]. Because organ dysfunction may limit dosing [15] and hydroxyurea may not reverse severe tissue injury, we recommend treatment before organ damage occurs [7].

Our study has limitations. Subjects were not followed from birth, and thus conclusions are limited to adults. Treatment duration is unknown, though a significantly higher HbF at first and last visits in the hydroxyurea group suggests extended treatment. Compliance and individual response to hydroxyurea were not monitored, and causality is not definitive due to the retrospective design. We are not able to assess whether hydroxyurea doses were lower as a result of organ damage. However, many patients without organ damage had been started and maintained on a dose less than the recommended dose for years. Further, since we are a tertiary referral center, the amount of observed organ damage could be over-represented. However, because SCD is an orphan disease, this study provides real world data with conclusions overlapping with prospective trials [32]. In summary, HbSS is still characterized by organ damage and early death despite improved hydroxyurea treatment. A proper hydroxyurea dose is associated with higher HbF, less organ dysfunction, and improved survival. Prospective trials of new indications for drugs that induce HbF are indicated to further evaluate beneficial effects on organ function and survival.
